# Occupational and Driving Challenges Within Sleep Medicine

**DOI:** 10.1111/jsr.70063

**Published:** 2025-04-09

**Authors:** Fran Pilkington‐Cheney, Walter T. McNicholas

**Affiliations:** ^1^ Department of Psychology, School of Social Sciences Nottingham Trent University Nottingham UK; ^2^ School of Medicine and the Conway Research Institute University College Dublin Dublin Ireland; ^3^ Department of Respiratory and Sleep Medicine St Vincent's Hospital Group Dublin Ireland

**Keywords:** driving regulations, obstructive sleep apnoea, safety, shift work, sleep health, sleepiness

## Abstract

Shift work is a necessity for a society that requires 24/7 services. However, working around the clock can cause a misalignment with our sleep‐wake cycle, resulting in sleepiness, impaired cognition and poor health. Due to the prevalence of shift work within safety‐critical contexts, there is a need to further understand the causes and consequences of non‐typical work on sleep, wellbeing, health and safety, as well as ways to effectively reduce this risk. Furthermore, disorders such as obstructive sleep apnoea, which is the most prevalent medical cause of sleepiness, compound the adverse health and safety consequences of shift work. This article provides an overview of some of the key occupational and sleep health challenges related to shift work: accurate measurement of sleepiness, mitigation, long‐term sustainability and work/life balance. We conclude by proposing four directions for future work in this area to consider, with the overall aim of improving the sleep, health and wellbeing of the shift‐working population.

## 1 Introduction

Our society requires individuals to be awake and perform tasks around the clock. Many shift workers, that is, those that work outside typical daytime hours (e.g., 8 am–6 pm) with a variation of work and schedules (Kecklund and Axelsson 2016), are not only required to be alert at times that are not aligned with their circadian rhythms, but also to perform safety‐critical tasks at all hours of the day. Occupational sleep medicine aims to mitigate the effects of sleep loss, time of day, and workload to improve safety and wellbeing in the workplace. However, shift work is a major challenge to this.

The timing of sleep and work can result in shortened and offset sleep opportunities, which in combination with other factors (e.g., exposure to light) can result in shift workers often experiencing shorter sleep of poorer quality (Yong et al. 2017) and impaired cognitive function (Chellappa et al. 2019). This is particularly relevant when thinking about safety‐critical tasks such as driving. Many shift workers drive, whether this be as professional drivers or to commute to/from work. As experiencing sleepiness when driving is associated with increased crash risk (Bioulac et al. 2017; Sprajcer et al. 2023), driver impairment is an important consideration in relation to shift work.

This, therefore, poses a critical societal issue: providing a 24/7 service while promoting good sleep health and a safe and sustainable work culture. This paper aims to consider some of the key challenges faced within shift work, in terms of the prevention, measurement, and management of sleepiness, as well as sleep disorders and safety risks, to suggest directions for future research.

### 1.1 Shift Work, Sleepiness and Safety

Shift work is not always conducive to good sleep health. Shift work can disrupt the regular sleep–wake cycle causing circadian misalignment, resulting in shortened, disturbed or irregular sleep, as well as exposure to light and food at the ‘wrong’ time (Baron and Reid 2014). This has several implications, with research showing the negative impact of circadian misalignment on health, wellbeing and safety (Kecklund and Axelsson 2016; Moreno et al. 2019; Rajaratnam et al. 2013), and more specifically, cognition (Chellappa et al. 2019), mood (Chellappa 2020) and sleepiness (Sallinen et al. 2005; Åkerstedt 1988). Consequences also include increased risk of health issues, such as cardiovascular disease (Morris et al. 2016a), impaired glucose tolerance (Morris et al. 2016b), diabetes (Gao et al. 2020), obesity (Chaput et al. 2023; Liu et al. 2018) and poor mental health (Brown et al. 2020). Additionally, many public sector occupations have increased exposure to stress or traumatic events, for example emergency services (Nguyen et al. 2023; Wild et al. 2016), which can further impact sleep, health and wellbeing.

There are also safety implications. Sleepiness in the workplace can result in accidents and greater risk of occupational injury (Rajaratnam et al. 2013; Uehli et al. 2014; Wagstaff and Lie 2011; Williamson et al. 2011). There are links between shift work, sleepiness, and work performance; for example, shift working nurses are more likely to report excessive sleepiness, sleep problems, and experience sleep‐related work errors (Gander et al. 2019). Individuals experiencing probable shift work disorder are also more likely to report workplace errors (Reynolds et al. 2021). There is likely an awareness by those doing shift work that sleepiness can impair performance, which may then impact safety (Booker et al. 2024), and that this risk can also occur after a shift during the commute (Booker et al. 2024). Commuting can extend the amount of wakefulness within a 24 h period (Mollicone et al. 2019) and can be particularly problematic if driving is involved.

#### 1.1.1 Driver Sleepiness

Driver sleepiness is a prevalent issue, contributing to approximately 15%–20% of road crashes globally (Hallvig et al. 2014; SWOV 2019; Zwahlen et al. 2016). Feeling sleepy behind the wheel is not uncommon in shift workers, with reports of sleepiness during professional driving (Miller et al. 2020; Onninen et al. 2020, 2021; Pylkkönen et al. 2015; Robbins et al. 2022a) and during the commute home (Anderson et al. 2018; Ftouni et al. 2012; Gander et al. 2019; Smith et al. 2020). The occurrence of sleep disorders is also common among professional drivers. For example, a large study of Finnish truck drivers estimated the prevalence to be between 28% and 40%, coupled with significant inability to stay awake in 1 in 20 drivers (Huhta et al. 2021). The prevalence of sleep disorders is an important issue within shift work, due to the associated risk with health and safety outcomes (e.g., Barger et al. (2015).

Obstructive sleep apnoea (OSA) is the most prevalent medical cause of sleepiness (Pérez‐Carbonell et al. 2022) and is an important risk factor for motor vehicle crashes (MVC) (Bonsignore et al. 2021). Patients with the disorder are 2–3 times more likely to have a MVC compared to the general driving population (Tregear et al. 2009), but effective therapy with continuous positive airway pressure (CPAP) has been reported to reduce this excess risk (Tregear et al. 2010). A report using data from the Swedish Traffic Accident Registry, indicated a MVC risk ratio of 2.45 in OSA patients compared to controls, which was reduced to control levels in patients who were compliant with CPAP (Karimi et al. 2015). Sleepiness at the wheel is the most important risk factor for MVC relating to OSA (Bioulac et al. 2017), which is reinforced by a recent report from France that also demonstrates the importance of compliance with therapy in MVC risk reduction (Coelho et al. 2024). Furthermore, one night's CPAP withdrawal results in a marked deterioration in simulated driving performance among OSA patients who were compliant with therapy (Filtness et al. 2012). Sleepy drivers with OSA often engage in efforts to limit the risk of MVC while driving such as opening the window, pulling over to rest, and taking a caffeinated drink such as coffee (Dwarakanath et al. 2024).

**FIGURE 1:**
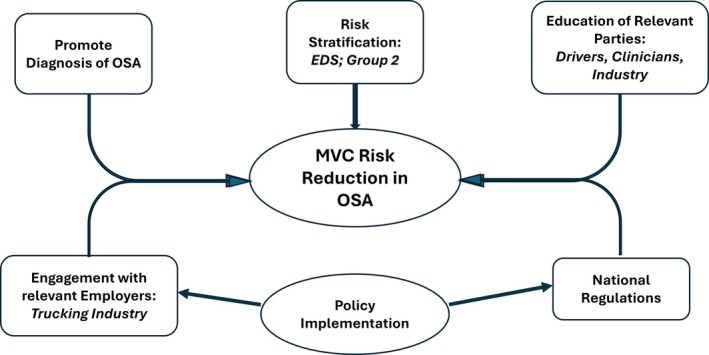
Strategies to improve the identification of at‐risk patients with OSA and their treatment. EDS, excesssive daytime sleepiness.

The MVC risk in patients with OSA is particularly important in shift‐working commercial drivers, especially long‐haul truck drivers, as these drivers usually drive very large vehicles for long hours on high‐speed motorways (Das et al. 2022; Fanfulla et al. 2021). Commercial drivers may also be exposed to additional driving situations that predispose them to increased sleepiness and MVC risk such as shift work, sleep restriction, and night driving. A report from the USA indicates that truck drivers with OSA who were adherent to CPAP therapy had an MVC risk similar to that of a general population of truck drivers, whereas nonadherent patients had a five‐fold higher crash rate (Philip et al. 2023). These findings underline the importance of identifying and treating patients with OSA in the expectation that this will result in a reduction in MVC rates in this patient population. This objective requires the identification of the OSA population at risk of MVC, education of all relevant parties including patients, clinicians, and employers, in addition to official driving policies to limit driving in symptomatic OSA patients, as already the case for other medical disorders such as epilepsy and cardiovascular disease (Figure 1).

### 1.2 Regulations

#### 1.2.1 Shift Work Regulations

Regulations relating to shift work and sleepiness (or sleep‐related fatigue) are complex and context dependent. There has previously been an overreliance on prescriptive rules around hours of work and rest, for example, within transportation a focus around driving hours, aimed to reduce time on task (European Parliament, Council of the European Union 2006). As described by Dawson et al. (2021) this can often be used as ‘proxy for fatigue management’. However, being led by hours of work may remove the focus on monitoring and management of risk related to sleep‐related fatigue, and result in increased levels of sleepiness going undetected. Although individuals have a responsibility to be ‘fit‐for‐work’ or in the least report when they are not, it is an employer's legal responsibility to manage sleepiness risk. As described by the UK Health and Safety Executive (HSE) (Health and Safety Executive 2017) ‘compliance with the Working Time Regulations alone is insufficient to manage the risks of fatigue’.

To some extent, the likelihood of when sleepiness may occur is predictable (thinking about time of day and shift duration), so therefore workplaces may establish a risk threshold about the level of sleepiness that would be acceptable (see Moslener et al. (2024) for overview). Recently, guiding principles have been published to help stakeholders make decisions relating to shift duration and its effects on performance, health and safety, informed by scientific evidence (Gurubhagavatula et al. 2021). Importantly within this is reference to pre‐existing policy frameworks which could be adapted to a variety of workplaces, emphasising a move towards risk‐based approaches to sleepiness management. Similarly, the HSE provides free guidance on managing shift work for a range of stakeholders, emphasising the legal duty to assess shift work risks and improve associated safety and ill health (Health and Safety Executive 2006).

#### 1.2.2 Driving Regulations for Patients With OSA

The recognition of increased MVC risk in patients with OSA raises the potential role of official regulations to limit driving in untreated patients with the disorder, especially in the context of the beneficial impact of CPAP on this risk. Over many years, expert groups in the field of sleep disorders such as the European Sleep Research Society (ESRS) and the European Respiratory Society (ERS) lobbied officials and politicians on the need for driving regulations in untreated patients with OSA (Krieger et al. 2002). In response to this advocacy, the European Commission established an official Working Group in 2012, which led to an official report that was accepted by the Commission (McNicholas et al. 2013) and resulted in a legal directive from the European Union (EU) in 2014 that set out criteria for patients with symptomatic OSA to continue driving (Bonsignore et al. 2016). Briefly, this directive specifies that patients with moderate or severe OSA, as measured by an apnoea‐hypopnoea index (AHI) > 15/h and associated sleepiness, should be restricted from driving until the disorder is effectively treated. Such EU Directives are mandatory for implementation in member states. This directive, which applies to a population of ~450 million, represents the largest population worldwide to be subjected to driving regulations for OSA.

Evidence is lacking in that official regulations regarding fitness to drive in patients with OSA result in a reduced MVC risk and further research is required to demonstrate benefit. However, a Swedish study comparing OSA patients treated with CPAP who were undergoing certification of fitness to drive with a general population of CPAP‐treated OSA patients reported a higher CPAP compliance and greater reduction in subjective sleepiness (Grote et al. 2018), suggesting an incentive to comply with treatment to retain driving certification (McNicholas 2018).

While no such regulations are applied in the USA at a federal level, the topic of enhancing public health and safety by diagnosing and treating OSA in the transportation industry has been the subject of a recent position statement by the American Academy of Sleep Medicine (Das et al. 2022). The statement urges key stakeholders, including clinicians and patients, to follow guidelines previously issued by a Medical Review Board of the US Federal Motor Carrier Safety that were not officially implemented. The statement also indicates that the current standard of care demands action to reduce the serious health and safety risks of OSA in commercial drivers. The available evidence indicates the potential benefit to driving safety from engagement with the trucking industry about the diagnosis and treatment of OSA and supports the need for a broad‐based education programme directed at the transportation industry.

## 2 Challenges for Sleep Medicine

Within developed countries, approximately one quarter of the workforce are shift workers (Wong et al. 2019). It is therefore vital to address issues such as these, to ensure a healthy and safe workforce. However, there are several challenges to this.

### 2.1 Accurate Measurement of Sleepiness Within Shift Working Environments

There is a substantial body of literature concerning the measurement of sleepiness within controlled, laboratory settings, and this has resulted in the establishment of several validated measures that can be useful in determining alertness levels. Although an electroencephalogram (EEG) can provide the most comprehensive information as to someone's alertness, it is not practical for ongoing measurement during shift work operations. Similarly, while the Multiple Sleep Latency Test (MSLT) (Carskadon 1986) and MWT (Mitler et al. 1982) are useful in determining sleepiness and wakefulness respectively, reports state they may be less accurate in the prediction of real‐life driving ability (Virtanen et al. 2019). A simpler variant of the MWT is the Osler Sleep Resistance test (Bennett et al. 1997). However, due to these tests being complex and time‐consuming, they are not practical for everyday use in applied settings.

Subjectively, certain scales are well‐used within the sleep and road safety literature. The first is the Karolinska Sleepiness Scale (KSS) (Åkerstedt and Gillberg 1990), a 9‐point scale ranging from 1 = ‘very alert’ to 9 = ‘very sleepy, fighting sleep, an effort to remain awake’. The KSS has been validated against physiological and performance measures (Kaida et al. 2006; Sagaspe et al. 2008; Åkerstedt and Gillberg 1990) and is relatively easy to administer, providing a level of sleepiness in understandable terms. The Epworth Sleepiness Scale (ESS) (Johns 1991) is also commonly used to assess daytime sleepiness, but unlike the KSS, does not provide repeated ratings of momentary sleepiness, albeit not continuous. The KSS has been frequently used to provide ratings of sleepiness during shiftwork (Miller et al. 2024; Onninen et al. 2020; Öster et al. 2024; Sallinen et al. 2017). Although a recent review concluded that drivers are aware of their own sleepiness when driving (Cai et al. 2021), shift workers generally may be reluctant to report sleepiness for fear of disciplinary consequences, and may under‐report or choose to continue working (Maynard et al. 2021). They may also face work or peer pressures, as well as lack of trust in the ‘system’, which may influence accuracy in measurement. Prolonged use, particularly in monotonous environments may also influence the reliability of the measurement.

Alertness, or lack of, can also be inferred through measures of performance. The Psychomotor Vigilance Test (PVT) (Dinges and Powell 1985) is popular for determining sustained attention and is particularly susceptible to sleep loss (Balkin et al. 2004). However, administering the test can disrupt work tasks, can take time (although shorter versions have been developed (Basner et al. 2011; Basner and Dinges 2012) and does not provide continuous measurement. If driving is concerned, then using vehicle metrics is another approach, although these could be susceptible to other factors (e.g., weather, distraction etc.).

Physiological and body measurement technologies can be incorporated into a variety of work environments using objective surrogates of sleepiness (e.g., eye blinking and lane deviation) which correlate well with conventional measures of sleepiness. This is highly relevant to in‐vehicle technology designed to detect drowsy driving (Howard et al. 2019). Within professional driving, cameras can be placed in front of the operator to provide information on various features (e.g., yawning, head and body movements etc.). Ocular activity is a well‐used measure, with indicators such as duration of blinks providing an estimation of sleepiness. The most well‐known indicator is Percentage of Eyelid Closure (PERCLOS; Dinges and Grace 1998, measuring the percentage of time the eyes are more than 80% closed. Although validated within lab, simulator, and on‐road settings (e.g., Cori et al. 2019; Sparrow et al. 2019) a recent review highlights issues, such as questions as to its effectiveness for detecting moderate sleepiness, the lack of research looking at use with patients with sleep disorders, inconsistent evidence for the efficacy of sleepiness detection after night shifts, and the multiple definitions used within the literature (e.g., sampling rate of blinks) (Abe 2023). This type of measurement can also be dependent on equipment, detection quality, and processing. Abe (2023) suggested the need to integrate PERCLOS detection with other metrics, with a multi‐modal approach also being suggested by others (Hasan et al. 2022; Tomson et al. 2025), although this needs further exploration. Importantly though, ‘fatigue‐detection’ technology is typically used to detect sleep onset (e.g., Dawson et al. (2014), which may be too late.

Research has recently focused on the use of heart rate, or heart rate variability (HRV), to detect sleepiness, particularly when driving (see Lu et al. (2022) for review). One of the benefits to this is the ease of use in real life settings (Lohani et al. 2019) overcoming some of the practical issues of daily use of physiological measures. HRV can be measured using chest straps or wrist bands and photoplethysmography (PPG), meaning it may be more feasible in a shift working context. However, as reported by (Lu et al. 2022), there is an inconsistency within the literature as to the accuracy for detecting sleepiness when driving, which may be due to the range of methodologies used (e.g., wide age ranges, sex differences, simulator vs. real road, impairment manipulation, how HRV was measured, ground truth employed) and small sample sizes. Although this measure has the potential for use in occupational contexts beyond driving, as well as being integrated into multi‐modal approaches, further research needs to be conducted to understand the relationship between sleepiness and HRV, particularly in complex, real world settings (e.g., at work, or when driving) where a range of factors may be present that would influence HRV.

A huge challenge to the accurate measurement of sleepiness is individual differences. There are inter‐ and intra‐individual differences in the sensitivity of sleepiness and consequences to this, which need to be accounted for. Sleep need, age, sex, chronotype, medical conditions and sleep disorders, personality, stress susceptibility—these factors can influence detection, prediction and effective countermeasures (Anund and Dahlman 2023) and are a challenge to any ‘one size fits all’ approach that may be deployed in operational contexts. Importantly, there is also interindividual variability in perception of impairment, particularly self‐appraised vigilance during night work (Boivin and Boudreau 2024). Accurate recognition of sleepiness is an important part of not only the effectiveness of self‐reporting, but also Fatigue Risk Management Systems (FRMS) (Sprajcer et al. 2022) and the decision to use countermeasures (Anund et al. 2015).

Much of the literature also focuses on shift working drivers, likely due to the safety risk of being impaired behind the wheel. Although relevant in some respects, further exploration of sleepiness measurement in non‐driving shift workers may mitigate the risk of sleepy driving via the commute as well as workplace injury and incidents. Detecting sleepiness in a vehicle can be challenging, however drivers are in a fixed position. This may be different to more ‘mobile’ shift workers, in which case subjective reporting or alertness testing may be more appropriate as reviewed by (Brossoit et al. 2023).

Ultimately, for accurate measurement of sleepiness to occur, the working environment must be considered, so there is a need for evaluation of methods in a range of situations and with varying levels of sleepiness. As well as issues related to data privacy and usage that need to be considered, there also needs to be engagement from the workforce. Creating an open safety culture, where sleepiness and sleep health can be discussed honestly, without fear of repercussions, would aid this; however, this takes time and requires commitment from all levels of an organisation.

### 2.2 Optimum Assessment of Sleep‐Disordered Breathing and Sleepiness

The use of AHI as the primary metric of OSA severity has been challenged and there is increasing emphasis on the use of alternative and/or additional measurements such as oxygen saturation and heart rate variability to evaluate the severity of the disorder (Pevernagie et al. 2020). Furthermore, AHI correlates poorly with sleepiness, which further limits the importance of AHI in assessing driving risk and emphasises the importance of including symptoms such as fatigue and sleepiness in the assessment of driving risk. This topic is rapidly developing and is the subject of the ongoing EU Horizon 2020‐funded research project “Sleep Revolution” (Arnardottir et al. 2022), which particularly focuses on the role of automatic analysis of relevant signals using machine learning tools.

The EU Directive specifies that sleepiness is required in addition to the diagnosis of OSA on a sleep study to judge the applicant's fitness to drive but does not indicate specific criteria for sleepiness and, in most cases, this assessment relies on the patient's subjective report of sleepiness. This reliance is problematic, as many OSA patients, especially professional drivers, may not admit to being sleepy because of concerns that such an admission may prevent them from driving. This aspect underlines the importance of appropriate education on the risks of untreated OSA and the benefits of successful treatment. A new scale, the BOSS scale, which specifically focuses on driving aspects, has recently been developed to better evaluate OSA‐related driving risk (Philip et al. 2023), but the scale still requires drivers to self‐report symptoms while driving, which has the same potential for under‐reporting, especially in professional drivers.

## 3 Mitigation of Sleepiness

Mitigating sleepiness within working environments is complex. There is a huge challenge in providing a continuous or productive service, which requires individuals to operate against their circadian rhythms, effectively manage sleepiness, consider additional influencing factors, and maintain reasonable costs. Approaches to this can be considered at an individual and operational level.

A report by the AAA (Bayne et al. 2022) identified six countermeasure classifications for driving, which broadly divide into individual (behavioural) and higher‐level operational countermeasures (technological, infrastructure, educational, medical and policy), many of which are applicable to shift work environments. Behavioural countermeasures are defined as strategies that an individual worker or driver may engage with either before, after, or during work to alleviate sleepiness and are typically reactive in nature. While caffeine and napping are the most effective behavioural countermeasures and well utilised (e.g., (Centofanti et al. 2018; Pylkkönen et al. 2015; Smith et al. 2020), it is commonly reported within the literature that strategies with little evidence of efficacy are used in working contexts to try to reduce sleepiness, for example, in medical professionals (Booker et al. 2024; Smith et al. 2020) and professional drivers (Filtness and Naweed 2017; Onninen et al. 2020; Pilkington‐Cheney et al. 2020). More research is needed to explore the reasoning for this; for example, it has previously been shown that the workplace can be a barrier to engagement with effective countermeasures (Pilkington‐Cheney et al. 2020).

From an operational perspective, strategies to mitigate sleepiness can include both proactive and reactive measures, for example interventions aimed at reducing the likelihood of sleepiness, and policies and procedures for managing sleepiness during shifts. Common evidenced‐based approaches can include shift scheduling, consideration of the work environment (e.g., lighting), access to countermeasures, as well as education programs (Caldwell et al. 2019). However, there is inconsistency in how these are applied within operational contexts, which often fail to consider the huge variation of shifts, occupations, personal responsibilities etc.

There is recent acknowledgement that advice needs to be tailored. Workers can often be encouraged to follow sleep hygiene guidance; however, as argued by Shriane et al. (2023), this can often be impractical for shift workers (e.g., maintaining a consistent sleep schedule), go against sleepiness advice (e.g., restricting caffeine) or not take into account some of the challenges shift workers face (e.g., transitioning from nights to days). While this group has worked towards establishing some shiftwork‐specific guidance with the help of experts in the field (Shriane et al. 2023), there are still challenges in terms of acceptance and feasibility of implementation, with limited knowledge as to the effectiveness on sleep outcomes.

Digital offerings may provide more flexibility, such as sleep ‘apps’ that are aimed at workers with varying schedules. These are being tested within different occupational contexts with the goal of trying to improve sleep health within shift workers (e.g., Counson et al. 2021; Murray et al. 2023; Robbins et al. 2022b; Shriane et al. 2024; Varma et al. 2024). However, many of these utilise self‐report measures of sleepiness and sleep, so may benefit from the future additions of objective measurements, as well as exploring feasibility of use within different industries. It may be that personalising advice or tailoring it to an individual roster will provide even further benefit to sleep, safety and health outcomes, although not an easy task for companies with large workforces and little sleep expertise.

There is also the issue of engagement. Although workers have reported a lack of guidance relating to managing sleep around shiftwork (Booker et al. 2024), some intervention studies have found differing levels of engagement with activities or tasks (e.g., Shriane et al. (2024) sometimes due to boredom (Varma et al. 2024). Optimisation is therefore important and potentially can be enhanced by involving shift workers in the design (Counson et al. 2021).

A high‐level intervention to aid in the identification, prevention and management of sleepiness and fatigue is a Fatigue Risk Management System (FRMS), replacing more outdated, prescriptive hours of work regulations Gander et al. (2017). FRMS are evidence‐based approaches (Lerman et al. 2012) which monitor ‘fatigue’ risk (typically sleepiness) to ensure workers are alert and safe when operating. FRMS systems are typically comprised of key components (Sprajcer et al. 2022) ranging from predictive (monitoring work schedules), proactive (real time worker fitness for duty) and reactive (identifying contribution to safety events). As noted by Sprajcer et al. (2022), many industries may implement components separately, perhaps alongside prescriptive rules, for example training and education or roster analysis. Although there is a lack of evaluation of whole FRMS systems, certain components such as biomathematical models, self‐report measures and performance monitoring do improve safety and fatigue metrics, therefore Sprajcer and colleagues argue that combining components should be similarly effective. Importantly, they identified some key barriers, many of which pose a challenge for any workplace sleep interventions, such as shared responsibility, buy in from all levels (including managerial and executive), and financial resource and time related costs.

But vitally to ensure the effectiveness of any intervention, there must be an understanding of the cause of the impairment. Fatigue and sleepiness are used interchangeably within the literature, but they are distinct. As described by May and Baldwin (2009), while sleepiness (or sleep‐related fatigue) is physiological and due to homeostatic and circadian factors, fatigue can be task related and include either active or passive fatigue. Ultimately both can lead to impairments in performance, but to be effective, the appropriate countermeasure must be chosen depending on the cause. While it is important to recognise that ‘fatigue’ can be a combination of factors (both task and sleep related), there needs to be greater transparency within the research in terms of defining ‘fatigue’ and how it is measured or manipulated, to aid in our understanding and ability to effectively manage it within operational contexts.

## 4 Longer Term Sustainability

Ensuring the safety and sustainability of the workforce is a huge challenge within shift working operations. As previously mentioned, shift work and circadian disruption are associated with adverse health effects, and not only is this a concern at an individual level, but also from an economic point of view.

Poor sleep and shift work have a detrimental impact on productivity (e.g., Folkard and Tucker 2003; Mulgrew et al. 2007), have been shown to be an independent predictor of sick leave, and poor physical and mental health (Doi et al. 2003), and could be a cost to employers and stakeholders. For example, OSA has been associated with increased daytime sleepiness, reduced performance and productivity, and increased healthcare costs (e.g., Léger and Stepnowsky 2020; Mulgrew et al. 2007) and insomnia has been associated with reduced presenteeism and increased absenteeism and risk of accidents or errors (Daley et al. 2009; Léger et al. 2006). Therefore, treating sleep disorders may have a positive benefit economically to employers (Wickwire et al. 2016, 2019). Specifically in relation to shift work, increased sickness has been shown in those working multiple (4+), consecutive night shifts (Ropponen et al. 2019).

As concluded by Glick et al. (2023), workers' sleep problems are related to the worsening of workplace outcomes and are associated with an increased cost, suggesting employers have an ‘economic stake’ in their employee's sleep. This stake is quite substantial. The cost of poor sleep has been estimated to range between $322 and $1967 per employee (Glick et al. 2023) with Hillman et al. (2018) suggesting an overall cost in Australia of $45.21 billion (2016–2017), made up of financial costs (e.g., healthcare, productivity, reduced employment, premature death) and non‐financial costs of reduced wellbeing.

In terms of safety implications, a recent review assessed the societal consequences of sleepy driving and found it not only increases the risk of a MVC but has an estimated direct cost of $139.4–$152 billion per year in the US and 43€–337€ billion (Léger et al. 2019). Driver sleepiness can also lead to impaired work ability, with an indirect cost of sleepy driving for lost human lives of $2372 billion worldwide (Léger et al. 2019).

Aside from poor sleep, there is also evidence to suggest that individuals who regularly work shifts also experience a change in health behaviours, which can contribute to reduced health and wellbeing. For example, eating light and food at the wrong time (Baron and Reid 2014), irregular eating and being exposed to unhealthy food choices (Lowden et al. 2010), being less physically active (Vandelanotte et al. 2015), binge drinking (Dorrian et al. 2017) and smoking (Härmä 2006), all of which may contribute to societal/economic costs (e.g., healthcare).

Interventions have been suggested for employees to identify and reduce sleep‐related problems; therefore, reducing costs. For example, introducing validated sleep measures, sleep disorder screening, and workplace sleep interventions (Glick et al. 2023). However, for sleep‐health interventions to be effective, there needs to be sustained engagement from employees and stakeholders, as well as consideration of individual differences, the industry and workplace, and feasibility within the constraints of the job.

### 4.1 Work/Sleep Balance

One of the biggest challenges shift work poses is balancing work and sleep, to ensure safe and alert workers who can engage with a healthy lifestyle. This requires individuals to ensure they are ‘fit‐for‐work’ and that their workplace ensures that this is achievable (e.g., adequate time between shifts, education about sleep and shift work etc.).

Within operational settings, there is the potential for a mismatch to occur between business pressure (e.g., getting the ‘work’ done) and safety (e.g., an alert workforce). Often individuals may report fatigue and sleepiness as illness to avoid any potential consequences (Maynard et al. 2021) but may also feel pressure to either continue working as they are nearly at the end of the shift, or to continue driving as they are nearly home (Smith et al. 2020). It has been highlighted that motivation to act is an important component in the decision to use countermeasures (Anund et al. 2015), and this is true for all stages of safe shift working, from obtaining adequate sleep, commuting to and from work, continuing work and reporting any impairment. Therefore, effort needs to be made to educate workers and develop cultures where motivation to act can be a positive decision (e.g., report, stop working and have a nap) rather than negative (e.g., push on and don't report).

Ensuring healthy shift workers also means recognition of their personal circumstances. Although shift workers may recognise benefits to shifts (e.g., flexibility and financial), these can be undermined by the costs (sleep deprivation, impact on family and social life) (Booker et al. 2024). Individuals working shift schedules at odds to family and society can face competing demands on their time between shifts, including sleep. However, a recent study reported that between the health behaviours of sleep, diet and physical activity, sleep was prioritised by shift workers, possibly due to the awareness of the ill effects of shift work, although this did not result in meeting sleep duration recommendations (Gupta et al. 2023). Research and potential interventions need to consider all aspects of a shift workers life when thinking about causes, consequences and mitigation of sleepiness and promotion of good sleep health. For example, individual differences are well reported within the sleep literature—as mentioned earlier, age, sex, chronotype, medical conditions and sleep disorders, as well as commuting distance, living arrangements, family and social commitments can all influence an individual's sleep, alertness and success in balancing sleep and work. These factors can sometimes be overlooked in interventions likely due to the complexity of including them, but, nevertheless, have a direct impact on the prevention and mitigation of sleepiness and poor sleep health.

## 5 Future Directions

### 5.1 Sleepiness Measurement and Effective Interventions

There is current focus on the development of interventions to help mitigate and manage sleepiness within shift workers, including apps, education programmes, as well as complete FRMS systems. However, there is a need for research to evaluate the effectiveness of these, as well as the feasibility in different contexts.

There is a strong need for more accurate and reliable objective measures of sleepiness that are suitable for implementation in large populations. This topic is a major research area and should be linked to developments in vehicle technology that are designed to detect impaired vigilance at the wheel such as lane drift and eyelid blinking (Howard et al. 2019).

Additionally, there is a need for more training and education for those that assess sleepiness risk in individuals with safety‐critical occupations, for example, physicians. Currently, the ESS is relied upon; however, due to its limitations (e.g., not being diagnostic nor measuring short‐term sleepiness fluctuations and as well as subjective bias), there is scope for the development of further tools or protocols to ensure accurate reflection of sleepiness risk when working. In terms of OSA, the present reliance on AHI criteria to categorise moderate and severe OSA may not be the most appropriate measure of severity and will likely require revision as new measures come into use.

From an individual perspective, the measurement and prevention of sleepiness need to account for individual differences (including lifestyle) and additional influences from a range of levels (from gender and age to policies and frameworks). Exploring enablers and barriers to engagement (e.g., motivation and behaviour change including psychological models) may also develop knowledge around effectiveness.

The differentiation between fatigue and sleepiness also needs further consideration, with research working to understand the relationship between the two and how this can be effectively mitigated. This is particularly relevant for shift workers with automated or monotonous tasks. As previously mentioned, transparency is needed as to what constitutes ‘fatigue’ within the research, as consistency in terminology or methodology would further our understanding of this complex phenomenon, particularly in terms of its relationship with the multitude of variables that can be encountered in the real world (e.g., stress, anxiety, temperature, etc.).

From an occupational point of view, as Sprajcer et al. (2022) note, there is a lack of evaluation into complete FRMS systems (rather than component parts) from a scientific and operational perspective. While FRMS systems have been successfully implemented within healthcare and the transport industry (Gander et al. 2011; Querstret et al. 2020), and more so out of the EU (Sprajcer et al. 2022) there is a need in other shift‐working industries such as construction, where (Zong et al. 2024) note best practice could be leveraged from established examples. The occupational context is an important factor in terms of feasibility, both in wider FRMS contexts and for specific interventions, and research is needed to explore how to effectively transfer scientific knowledge to a range of workplace settings, each with their own specific barriers. Identifying and addressing barriers to engagement, for example in terms of effective countermeasure use, is an important component of sleepiness mitigation. Finally, as workplace culture and shared responsibility are key enablers in terms of the success of an intervention (such as FRMS, Sprajcer et al. (2022) research should focus on how an open safety culture can be achieved, the barriers to this and how to address them, as well as exploring the effectiveness in terms of promoting good sleep health and reducing sleepiness.

Further studies are required to assess the efficacy of official policies such as the EU Directive in reducing MVC among patients with OSA who are effectively treated. Ethical considerations would likely prevent a RCT of CPAP in OSA to examine MVC rates, especially in sleepy patients. However, population studies based on clinical and accident registry data may facilitate studies of MVC rates before and after the introduction of such policies.

### 5.2 Attention to All At‐Risk Groups

There is a huge array of occupations that involve shift work. However, there is a tendency within the literature to focus on certain groups (e.g., medical professionals and transportation professional drivers). While this is important, many of these groups are also regulated and under more structured ‘monitoring’ via policies, procedures etc. Therefore, more diverse at‐risk groups of shift workers should also be given attention (e.g., gig economy and self‐employed workers, taxi drivers, contractors and student shift workers). This is not an exhaustive list, but groups such as this typically go ‘under the radar’ and face fewer restrictions, even from a prescriptive‐system point of view. However, they are not immune from sleepiness and fatigue effects, with many likely also engaging with regular driving (either for work or commuting), putting themselves and other road users at risk.

### 5.3 Technological Advancements

Despite huge scope to incorporate technology into the measurement of sleepiness during work and promotion of sleep health, there are still areas that need further development to ensure effectiveness, for example, improvements in accuracy. Research will be further enhanced in this area by a push for consistent methodologies, for example surrounding terminology, manipulation, ground truths etc. and the need for more real‐world testing of detection technology (Lu et al. 2022).

There may be scope for personalisation to help account for individual differences. This is important for both prediction and proactive prevention of sleepiness at work. However, each workplace will have its own challenges, so there is a need also for tailored approaches to different occupations.

Recently, work has been undertaken to develop ways to detect sleep deprivation using metabolomic biomarkers (Jeppe et al. 2024) or salivary metabolomics (Xu et al. 2019). Mainly focused on drivers, this is an interesting direction of research that needs more attention, particularly in terms of implementation and operational and legal implications.

Finally, with the move towards increasingly automated systems and vehicles, there is concern about the prevalence of fatigue (e.g., Ahlström et al. 2021; Körber et al. 2015; Vogelpohl et al. 2019), particularly as driving metrics will no longer be able to be used to detect impairment. More work needs to be done to understand the relationship and interaction between fatigue (passive, task‐related) and sleepiness in terms of measurement, prevention, and on‐shift countermeasures.

### 5.4 The Shift Work Lifespan

There is a body of research detailing the detrimental impact of shift work on sleep, health and wellbeing. But with methodological advancements, there is opportunity for more substantial longitudinal data collection to further understand the effects of shift work, sleep loss and circadian disruption over time, including recovery from these effects after post‐shift working, and the impact of interventions such as training and education (James et al. 2024). This would also be beneficial in terms of developing a body of research across all stages of the menstrual cycle and phases of the menopause to further our understanding of women's health.

The transition to shift work could also be further understood, considering how factors may protect workers (e.g., shift work tolerance including individual variability, resilience and fitness), or lead to the development or worsening of health/wellbeing issues (e.g., anxiety, depression, burnout and migraine), and how this can be effectively managed. Early intervention (sleep disorder screening, management) for those beginning shift work or who will experience shift working in the future may reduce poor health and safety outcomes, although there is a need for further randomised controlled trials to explore effectiveness (Brown et al. 2024).

With the development of interventions (e.g., educational apps), there is also a need to look at the longitudinal effects of these on shift worker sleep and health, whether some of the well‐known implications can be mitigated, or whether there is a need for a ‘stepped’ approach to ensure continued effect.

## 6 Conclusions

Shift working is vital for a society that requires 24/7 services. Nevertheless, it poses a challenge to occupational sleep medicine from health, wellbeing, and safety perspectives. Although efforts have been made to try to understand the causes of poor sleep and health, particularly in relation to sleep disorders, as well as the consequences of shift work, there is still much to be done. It is unlikely that sleepiness and fatigue and the occurrence of sleep disorders will be eliminated from shift work operations, due to the timing and nature of the work, but future research can continue to lessen the detrimental impact on sleep, health, and safety and improve the wellbeing of shift workers. Vitally, tailored investigations and interventions that consider the operational context and the individual are needed to drive this forward.

## Author Contributions

**Fran Pilkington‐Cheney:** conceptualization, writing – original draft, writing – review and editing. **Walter T. McNicholas:** conceptualization, writing – original draft, writing – review and editing.

## Conflicts of Interest

The authors declare no conflicts of interest.

## Data Availability

Data sharing not applicable to this article as no datasets were generated or analysed during the current study.
